# Does Race or Ethnicity Modify the Impact of Age on Cognition in Middle- and Older-Aged Adults?

**DOI:** 10.1093/geroni/igaa057.2825

**Published:** 2020-12-16

**Authors:** Indira Turney, Miguel Arce Rentería, Patrick Lao, Adam Brickman, Jennifer Manly

**Affiliations:** 1 Columbia University Medical Center, New York, New York, United States; 2 Columbia University, New York, New York, United States; 3 Columbia University Medical Center, new york, New York, United States

## Abstract

We compared verbal list learning, verbal memory, animal fluency, and letter fluency in 1407 education-matched participants from two community-based, intergenerational studies of cognitive aging and dementia. WHICAP participants are sampled from Medicare-eligible people aged 65+ and the Offspring cohort includes their middle-aged children. WHICAP participants (n=1218) were 72.1±6.5 years old and Offspring participants (n=189) were 53.7±8.4 years old at baseline. WHICAP participants had lower scores on most cognitive measures than Offspring participants; however, these differences were not uniform across race/ethnicity. Compared to non-Hispanic Whites, non-Hispanic Blacks in WHICAP had disproportionately lower scores on letter fluency compared to their offspring. On delayed verbal memory, non-Hispanic White and Hispanic offspring obtained higher scores than the parent generation – but among Blacks, memory scores were relatively low regardless of cohort. Racial disparities in cognition are apparent in both mid- and late-life and may be amplified in older age, particularly in Blacks.

